# Cognitive Performance in Patients with Systemic Lupus Erythematosus Using the Ped-ANAM

**DOI:** 10.3390/cells11244054

**Published:** 2022-12-15

**Authors:** Jaqueline Cristina de Amorim, Samara Rosa Sepresse, Jéssica Fernandes Vivaldo, Paulo Rogério Julio, Simone Thiemi Kishimoto, Roberto Marini, Paula Teixeira Fernandes, Lilian T. L. Costallat, Simone Appenzeller

**Affiliations:** 1 Post-Graduate Program of Child and Adolescent Health, School of Medical Science, University of Campinas, Campinas 13083-970, Brazil; 2 Laboratory of Autoimmune Diseases, School of Medical Science, University of Campinas, Campinas 13083-970, Brazil; 3 Medical Pathophysiology Post-Graduation Program, School of Medical Science, University of Campinas, Campinas 13083-970, Brazil; 4 Pediatric Rheumatology Unit, Department of Pediatrics, University of Campinas, Campinas 13083-970, Brazil; 5 Department of Sport Sciences, Faculty of Physical Education, University of Campinas, Campinas 13083-851, Brazil; 6 Department of Orthopedics, Rheumatology, and Traumatology, School of Medical Science, University of Campinas, Campinas 13083-970, Brazil

**Keywords:** neuropsychiatric lupus, neuropsychiatric manifestations, systemic lupus erythematosus, cognitive impairment, automated pediatric neuropsychological assessment metrics

## Abstract

Computerized batteries have been widely used to investigate cognitive impairment (CI) in patients with SLE. The aim of this study was to evaluate the cognitive performance of patients with SLE in relation to healthy controls using the Pediatric Automated Neuropsychological Assessment Metrics (Ped-ANAM) battery. In addition, we aimed to examine differences in Ped-ANAM scores according to age of disease onset, presence of disease activity, and disease damage. We included 201 consecutive adult-onset (aSLE) and childhood-onset SLE (cSLE) patients who were being followed at the hospital’s rheumatology outpatient clinic and 177 healthy controls. We applied the percentage of correct answers on the Ped-ANAM subtests and the Performance Validity Index (PVI) metric and correlated them with the Systemic Lupus Erythematosus Disease Activity Index (SLEDAI) and Systemic Lupus Erythematosus Damage Index (SDI). Then, we established their relationships with neuropsychiatric systemic lupus erythematosus (NPSLE). We observed CI in a total of 38 (18.9%) SLE patients and 8 (4.5%) healthy controls (*p* < 0.001). CI was observed in eight (19.5%) cSLE patients and 32 (20%) aSLE patients (*p* = 0.8175). Individual analysis of the aSLE subtests showed a significant difference in all subtests compared to healthy controls; the greatest differences were in matching to sample (*p* < 0.001) and memory search ( *p* < 0.001). In the cSLE group, we observed a difference in the code substitution subtests (*p* = 0.0065) compared to the healthy controls. In the evaluation of clinical outcomes, disease activity was significantly correlated with CI in cSLE (r = 0.33; *p* = 0.042) and aSLE (r = 0.40; *p* = 0.001). We also observed an association between disease activity and neuropsychiatric manifestations (*p* = 0.0012) in aSLE. In conclusion, we determined that cognitive dysfunction, mainly in memory and attention, was more prevalent in patients with SLE. In both the cSLE and aSLE groups, disease activity was associated with worse cognitive function. This is the first study to use the Ped-ANAM in Brazil. Longitudinal studies are necessary to determine how the Ped-ANAM will perform over time.

## 1. Introduction

Systemic lupus erythematosus (SLE) is a chronic systemic autoimmune disease [1]. Neuropsychiatric manifestations (NPSLE) are frequently observed and are among the most challenging presentations in clinical practice [2,3]. Cognitive impairment (CI), which is defined as a cognitive decline from a previous level of mental functioning documented by neuropsychological assessments, has an especially negative impact on quality of life and employment [4]. The validated American College of Rheumatology Neuropsychological Battery (ACR-NB) and Childhood Arthritis and Rheumatology Research Alliance (CARRA) battery are considered the gold standards for CI assessment in SLE [5,6,7,8]. However, their evaluation depends on qualified personnel, and the test durations range between 2 and 3 h [9,10]. In addition, not all subtests are validated in different languages, and a learning curve is observed with repetitions over time [9,10]. Thus, these batteries are expensive, time-consuming, and not easily accessible during daily clinical practice [9,10].

Cognitive assessments have followed the advancement of technology, and, by now, computer-based tests have been validated for use in general populations and in patients with chronic diseases [10]. Unlike their paper-and-pencil counterparts, computer-based measurements place less demand on the examiner in terms of time. However, there are several important issues involved in administering computer-based measurements that must be considered to ensure a reliable and valid assessment, such as automation in correction, ease of application, interest of the candidate in digital stimuli, self-administration flexibility, and the ability to perform the tests on a mobile device [9,10,11].

An example of a computerized battery for screening cognitive function is the Automated Neuropsychological Assessment Metrics (ANAM) test, a self-administered computer-based battery of tests that assesses neurocognitive efficiency [12]. It was developed by the US military to evaluate cognitive function in adults [13,14]. The most important advantages of the ANAM are speed, efficiency, and lack of need for special equipment since it can be performed on a standard computer. Furthermore, there is no learning effect, so it can be repeated multiple times in short time intervals [12,15].

The battery of tests takes 30–45 min to complete and includes a variety of tasks designed to assess measures of response time and accuracy [12,15,16,17]. Most ANAM tasks resemble commonly used neuropsychological tests but have been modified to require a relatively simple subject–computer interface in which the required answers are yes/no or equal/different discrimination, indicated by pressing one of two buttons on the mouse [12,15,16,17].

The Pediatric Automated Neuropsychological Assessment Metrics (Ped-ANAM) instrument is similar to the adult version, but has been modified to ensure that the test instructions and stimuli are age-appropriate and take cognitive development into account (e.g., lowered reading ability, simplified stimuli, etc.) [17,18]. Subtests include measures of simple reaction time, procedural reaction time, learning code and memory replacement, logical reasoning, spatial processing, continuous performance (sustained attention), math processing, paired grids, comparison to sampling, and Sternberg’s memory scanning paradigm [12,13,17].

The battery was recently translated and validated in Portuguese (Brazil). This is the first study to evaluate CI in patients with SLE using the Ped-ANAM [18]. Thus, the objectives of this research were to evaluate the performance of the Ped-ANAM as a screening tool for CI in patients with SLE, to identify differences between childhood-onset SLE (cSLE) and adult-onset SLE (aSLE), and to determine the association of disease activity and damage on Ped-ANAM performance.

## 2. Materials and Methods

### 2.1. Participants

The study was carried out in Campinas, Brazil. The convenience sample consisted of a total of 378 participants. Two hundred and one consecutive patients with SLE were recruited from the adult and pediatric rheumatology outpatient clinics at the University of Campinas. The definition of cSLE was disease onset before the age of 18 [19]. SLE patients were invited to participate in the study on the day of their routine medical consultations. The inclusion criteria were age 9 years or older and literacy in Portuguese as their native language. We excluded participants with other autoimmune diseases, neurological manifestations not related to SLE that could affect cognitive testing, and non-corrected vision problems that could prevent individuals from adequately viewing the stimuli on the computer. For the healthy controls, family members and friends of the participating patients were invited, as well as individuals without autoimmune diseases from the local community. In order to determine if there were differences according to age of disease onset, we compared cSLE patients with aSLE patients. A total of 177 healthy controls were included. For group comparisons, we classified healthy controls into two groups: ≤18 years and >18 years. 

### 2.2. Ped-ANAM

The following eight subtests are always performed by the software in the same order: simple reaction time, code substitution, logical relations, spatial processing, mathematical processing, matching grids, matching to sample, and memory search [12,15,16,17,18]. The number of stimuli in each subtest varies according to the activity. Upon conclusion of the test, the Ped-ANAM results are generated individually and automatically by the software. Each subtest provides metrics such as number of errors, successes, lapses, and performance speed [12,15,16,17,18]. In addition, the software also provides a result called the Performance Validity Index (PVI) based on a distributional analysis of the data [20]. This index is constructed from the weighted results of each subtest associated with the accuracy discrepancy and reaction time scores for each test. Weighted scores are assigned based on scoring frequency.

The PVI provides an indicator of whether the participant’s performance was within the expected range for someone with good effort. The score ranges from 0 to 33. By default, the reference group is an outpatient sample with a cut-off point of 14; 0–13 is defined as good performance exertion. Thus, lower results indicate good performance, and higher results indicate signs of possible CI. The Ped-ANAM battery is a screening tool; all results generated or combinations of scores may indicate CI [12,16,18].

### 2.3. Clinical Outcomes

Disease activity was assessed using the Systemic Lupus Erythematosus Disease Activity Index-2000 (SLEDAI) [21], and cumulative damage was determined using the Rheumatology Damage Index in patients with systemic lupus erythematosus (SDI) [22]. In addition, we reviewed medical records to verify the history of NPSLE according to the American College of Rheumatology (ACR) criteria [5,23].

### 2.4. Study Design

This quantitative, observational, descriptive, cross-sectional study was conducted in Campinas, Southeastern Brazil, to assess cognitive function in SLE patients using the Ped-ANAM.

### 2.5. Procedures

The project was approved by the local ethics committee (CAAE 39750914.3.1001.5404). All participants were personally contacted, informed about the study, invited to participate, and asked to voluntarily sign an informed consent form. A trained psychologist administered the Ped-ANAM to all participants in an individualized environment without interruptions. The computerized cognitive assessment lasted on average 35 min (median 37 min; range 30–50 min).

### 2.6. Statistical Analysis

Statistical analyses were performed in R version 4.0.3. Core Team R: A language and environment for statistical computing. R Foundation for Statistical Computing (Vienna, Austria, 2021). URL: https://www.R-project.org, accessed on 5 February 2022, [24]. Descriptive statistics and their distributions were computed. Data normality tests were performed. Sociodemographic characteristics were computed. A level of statistical significance of *p* < 0.05 was adopted. 

#### 2.6.1. Subtest Analysis

The descriptive results of the percentage of correct answers of the participants in each Ped-ANAM subtest were analyzed, and correlations with the control group were analyzed using the Kruskal–Wallis test.

#### 2.6.2. Performance Validity Index

The prevalence of CI was calculated using the Performance Validity Index (PVI) results. We also used the metric to compare performance between the patient group and the healthy control group using the Mann–Whitney U test.

#### 2.6.3. Performance Validity Index and Clinical Outcomes

The patients’ characteristics were summarized through descriptive analyses. Spearman’s correlation was used to determine whether the PVI scores were associated with disease activity (SLEDAI) and damage (SDI) in adult- and childhood-onset SLE. We also analyzed the performance of patients in the PVI metric based on the absence or presence of neuropsychiatric manifestations as described in medical records.

#### 2.6.4. Cognitive Score Performance

Finally, we performed principal component analysis (PCA) to calculate the Cognitive Performance Scale (CPS) score [25]. During PCA, the variance–covariance matrix of the (normalized) accuracy scores placed the Ped-ANAM subtests into a series of eigenvectors with corresponding eigenvalues. Each eigenvalue as a linear variation of all test accuracy scores was weighted by the values contained in the corresponding eigenvector. To preserve most of the total variance of the variance–covariance matrix, the first eigenvector was used in the CPS-PCA derivation.

## 3. Results

### 3.1. Sociodemographic Characteristics

We included a total of 378 participants: 201 consecutive SLE patients (183 [48.4%] women; median age = 28 years; age range = 9–76 years) and 177 healthy controls (124 [32.8%] women; median age = 22; age range = 9–60 years) (Table 1). When subdividing according to age, 104 individuals were ≤18 years (41 cSLE patients and 63 healthy controls), and 274 were >18 years (160 aSLE and 114 healthy controls). The demographic characteristics of these subgroups are presented in Table 2.

### 3.2. Subtest Analysis

When we analyzed the percentage of correct answers in each subtest for all participants, we observed the highest percentage of correct answers in the procedural reaction time subtest (96%) and the code substitution subtest (97%) (Table 3). The subtests with the lowest success rates were matching to sample (78%) and spatial processing (88%). The Kruskal-Wallis test (*p* = 0.001) revealed that there was a difference in the percentage of correct answers between the subtests, suggesting a difference in the content of each of the subtests.

We subdivided the descriptive data of percentage of correct answers for cSLE (Table 4) and aSLE (Table 5) patients. In the cSLE group, we observed that logical relations (mean correct answer = 97%; SD = 0.05) was the subtest with the highest percentage of correct answers, followed by the subtest simple reaction time (mean = 95%; SD = 0.09). The subtests with the lowest percentages of correct answers were matching to sample (mean = 78%; SD = 0.20), followed by memory search (mean = 80%; SD = 0.16). In the aSLE group, the subtests with the highest percentages of correct answers were logical relations (mean = 95%; SD = 0.047) and simple reaction time (mean = 95%; SD = 0.09). The lowest percentages of correct answers was matching to sample (mean = 85%; SD = 0.20). When compared to healthy controls, cSLE patients had worse results in memory search (*p* = 0.2733) and code substitution (*p* = 0.0065) (Table 4). When compared to healthy controls, aSLE patients had worse results in matching to sample (*p* < 0.001) and memory search (*p* < 0.001) (Table 5).

### 3.3. Performance Validity Index

The PVI descriptive results for all participants and according to age of disease onset are presented in Table 6. In the total group of all SLE patients, we observed a median of 7 (range = 0–33), and for healthy controls, a median of 4 (range = 0–22) (*p* < 0.001). Subdividing the results according to age of disease onset, we observed a median PVI in cSLE of 7 (range 0–23) and a median of 5 (range 0–18) in healthy controls (*p* = 0.0236). In aSLE, we observed a median of 7 (range 0–33), and for healthy controls, a median of 2 (range 0–22; *p* = 0.0108). Considering the cut-off value of 14 for the PVI metric, we observed that 38/201 (18.90%) patients with SLE and 8/177 (4.5%) healthy controls had low-effort results, suggestive of CI (*p* = 0.035). In addition, upon subdividing according to age of disease onset, we observed that 8/41 (19.5%) cSLE patients and 32/160 (20%) aSLE patients had CI (*p* = 0.8175). 

### 3.4. Performance Validity Index and Clinical Outcomes

We observed a correlation between the PVI and SLEDAI in cSLE (*r* = 0.33; *p* = 0.042) and aSLE (*r* = 0.40; *p* = 0.001). We also observed an association with neuropsychiatric manifestations (*p* = 0.0012) in aSLE patients (Table 7).

### 3.5. Cognitive Performance Scale

In the Ped-ANAM Cognitive Performance Scale, according to the principal component analysis reproduction method, the analysis resulted in 10 components, in order of the highest eigenvalue to the lowest eigenvalue. We used only the first component, with an eigenvalue = 2.6.

## 4. Discussion

Cognitive impairment is common in SLE patients and is one of the 19 neurological and psychiatric syndromes of SLE proposed by the American College of Rheumatology (ACR) [5,6,24]. Different studies have found different levels of CI because of the diversity in methods and neuropsychological tests used [26]. A systematic review determined that the prevalence of CI in patients with SLE based on a formal battery applied by a psychologist was 38% (ranging from 15 to 79%), while it was 26% (ranging from 12 to 42%) using a computerized instrument (ANAM) [26]. A formal battery was validated using the ACR-NB as the standard battery for CI evaluation, but it is expensive and time-consuming [26]. On the other hand, computerized investigations and screening tools can facilitate clinical practice at a lower cost and with greater ease of application [18,19,20,26]. Currently, the use of computerized batteries in the clinical and research contexts is becoming more common [26]. In the present study, the Ped-ANAM battery, which belongs to the set of virtual libraries of the ANAM battery, was used.

In their different versions, ANAM batteries have been used to screen for CI in diseases such as fibromyalgia, multiple sclerosis, Alzheimer’s disease, and Parkinson’s disease, as well as in those with substance abuse disorders, and after exposure to radiation. [27,28,29,30]. The Ped-ANAM is an adapted version of the ANAM battery. Changes were made to the test instructions to facilitate understanding by children as young as 9 years of age. Previous studies with English speakers used the Ped-ANAM to screen for CI in patients with cSLE [9,12,15,16].

This is the first study on the use of the Ped-ANAM in the Brazilian population after the translation of the battery into Brazilian Portuguese [18]. From the result metrics automatically generated by the Ped-ANAM, we first chose the percentage of correct answers in each subtest. Subsequently, to measure the presence or absence of CI, we used the PVI, which is a weighted method automatically generated by the software. We observed a high percentage of correct answers in all subtests, which was also observed during the translation and validation process and in previous studies [9,12,16,18,25]. A low frequency of correct answers is atypical and may suggest intentionally poor performance [9,18,25].

In our study, the correlation of the correct answers in the aSLE group showed significant differences in relation to the control group for all subtests. In cSLE, a difference between patients and controls was observed in the code substitution subtest (*p* = 0.0065). This difference may be associated with the number of participants in each group. Previous studies have shown that differences between SLE patients and healthy controls are mainly related to memory and attention [9,12,16].

In our study, according to the PVI, the data showed significant differences between the patient scores and the scores of the healthy controls. Other studies of the PVI have used a healthy university sample with known adequate effort (ANAM-PVI = 0.96). An outpatient brain injury sample with known adequate performance validity had significantly higher ANAM-PVI scores than healthy controls (ANAM-PVI = 3.83). These findings provide good support for using the PVI metric [20].

Previous studies validated a metric named the Cognitive Performance Scale (CPS) in cSLE [15,25]. In our results, we calculated the CPS score using principal component analysis of patient outcomes and obtained an accuracy coefficient of −1, indicating that it has a negative weight on PC1. Calculating PC1, we obtained a measure that varied between 1.13 and 3.96, which is much higher than the original study that validated the metric (0.25) [25]. This difference may be due to the demographics of the study populations, including language and education levels. More studies need to be performed to establish a cut-off point for our population [25].

Using the ACR-NB, cognitive dysfunction was observed mainly in visuospatial processing, working memory, short-term memory, visual perception, attention, associative learning, information processing speed, and spatial processing [6]. Frequently, more than one cognitive function is affected in SLE, indicating that there are multifactorial individual phenotypes [6]. SLE patients have considerable difficulties in verbal and non-verbal learning and working memory, followed by deficits in simple attention and psychomotor speed [26]. Patients often refer to their cognitive difficulties as “brain fog”, a term that describes the experience of CI in everyday life [24,31]. In our study, the main cognitive functions affected were attention and memory.

In our study, we observed that the prevalence of CI in all SLE patients was 18.90%, which is within the wide range of SLE CI rates (15–79%) described in the literature. The CI rate for the healthy controls in our study was 4.5% [26,30,32]. Compared to other studies that used the Ped-ANAM, we observed a CI prevalence of 19.5% in cSLE. This frequency is similar to previously observed results that ranged from 22.5 to 35% in cSLE [9,15,27].

Compared to the Ped-ANAM, the ANAM battery has been used more frequently in SLE studies [9,12,18,28,32,33,34,35,36]. A systematic review showed a pooled prevalence of CI of 39% [26]. In studies using the Mini-Mental State Examination (MMSE) and the Montreal Cognitive Assessment (MoCA), the CI prevalence was 11.1% based on the MMSE and 61.1% based on the MoCA [36,37]. The two batteries (MMSE and MoCA), as well as the ANAM batteries, are screening tools for cognitive function rather than diagnostic tests [35,36,37]. The assessment of CI through traditional batteries observed a prevalence ranging from 15% to 79% [26]. Studies prior to the 2000s had the highest prevalence of CI (38%); the prevalence decreased over the following decades to 34% (studies from 2000 to 2009) and 27% after 2010 [28]. The differences in the frequency of the CI results are due to different research batteries and methods.

Assessment of CI in SLE is not standardized, and attribution of CI to SLE can be difficult, as medications, infections, metabolic disorders, and hypertension can also cause CI [31,36,37]. CI has been associated with a variety of clinical and laboratory features, such as disease activity, antiphospholipid antibodies, presence of neuropsychiatric manifestations, pain, depression, and anxiety [31,36,37]. In our study, disease activity and NPSLE were associated with CI in aSLE [27,28,29,30].

This study has some limitations. One possible limitation is that the group of patients and the group of healthy controls differed in age and education level, which may have influenced the results. In fact, the high education level, which is known to impact cognitive ability, of our control group is worth noting. Another limitation is that cumulative corticosteroid dose can influence CI. We were not able to retrieve corticosteroid dose information for SLE patients, so we could not analyze this variable in relation to CI.

In conclusion, we determined that the Ped-ANAM is a useful instrument for the cognitive evaluation of SLE patients in Portuguese (Brazil). Cognitive dysfunction was more prevalent in SLE patients when compared to healthy controls, especially memory and attention. Future studies are needed to replicate other combinations of metrics and other associations with the participants’ profiles, thus increasing the knowledge of battery use for this population.

## Figures and Tables

**Table 1:** Sociodemographic characteristics.

Characteristics		SLE Patients (N = 201)	Healthy Controls (N = 177)	*p*-Value
		N (%)	N (%)	
Sex	Female	183 (48.4%)	124 (32%)	0.067
	Male	18 (4.8%)	53 (14%)	
Age	Min–Max	9–76	9–60	<0.001 *
	Median	28	22	
Education level	Elementary school unfinished	36 (9.5%)	40 (10.6%)	
	Elementary school finished	17 (4.5%)	2 (0.5%)	<0.001 *
	High school unfinished	19 (5.0%)	23 (6.1%)	
	High school finished	90 (23.8%)	22 (5.8%)	
	Technical education	6 (1.6%)	4 (1.1%)	
	University unfinished	17 (4.5%)	37 (9.8%)	
	University finished	15 (4.0%)	36 (9.5%)	
	Postgraduate	1 (0.3%)	1 (0.3%)	

SLE, systemic lupus erythematosus; Sex: the results indicated that the two groups were associated in this variable; * *p*-Value < 0.001; Age: SLE patients (median = 28 and interquartile range = [21, 41]) had a wider age distribution than healthy controls (median = 22 and interquartile range = [15, 29]); * *p*-Value < 0.001; Education: the results indicated the healthy controls’ possibly higher level of education.

**Table 2:** Sociodemographic characteristics of the total sample divided by childhood-onset and adult-onset SLE.

Characteristics		cSLE Patients (N = 41)	Healthy Controls (N = 63)	*p*-Value	aSLE Patients (N = 160)	Healthy Controls (N = 114)	*p*-Value
Sex	Female	36 (34.6%)	48 (46.2%)	0.234	147 (53.6%)	76 (27.7%)	<0.0001 *
	Male	5 (4.8%)	15 (14.4%)		13 (4.7%)	38 (13.9%)	
Age	Mean	12.9 (SD = 3.3)	14.6 (SD = 289)	0.0049 *	36.1 (SD = 12.6)	29.9 (SD = 10)	<0.0001 *
Education level	Elementary school unfinished	23 (22.1%)	36 (34.6%)	0.025	16 (5.9%)	5 (1.8%)	<0.0001 *
	Elementary school finished	7 (6.7%)	19 (18.3%)		11 (4.0%)	2 (0.7%)	
	High school unfinished	8 (7.7%)	2 (1.9%)		12 (4.4%)	4 (1.5%)	
	High school finished	0 (0.0%)	5 (4.8%)		84 (30.9%)	20 (7.4%)	
	University unfinished	3 (2.9%)	0 (0.0%)		1 (0.4%)	1 (0.4%)	
	University finished	0 (0.0%)	0 (0.0%)		17 (6.2%)	33 (12.1%)	
	Postgraduate	0 (0.0%)	0 (0.0%)		20 (7.4%)	18 (6.6%)	

cSLE Patients, childhood-onset SLE; aSLE Patients, adult-onset SLE; Age: cSLE had a wider age distribution than healthy; aSLE had differences in distribution compared to healthy controls for all variables, Age *p*-Value < 0.0001 *, Sex *p*-Value < 0.0001 *, Education level *p*-Value < 0.0001 *.

**Table 3:** Descriptive analysis of the percentage of correct answers of all participants in each Ped-ANAM subtest (N = 378).

Subtest	Min–Max (0–100%)	1Q–3Q	Median (0–100%)
Simple reaction time	0–100	60–100	85
Matching to sample	30–100	62–95	85
Spatial processing	45–100	85–95	90
Memory search	0–100	87–100	93
Mathematical processing	35–100	90–100	95
Matching grids	40–100	90–100	95
Code substitution	21–100	94–99	97
Logical relations	50–100	95–100	97

Ped-ANAM, Pediatric Automated Neuropsychological Assessment Metrics; Min–Max, minimum and maximum of correct answers; 1Q, first quadrant, 3Q, third quadrant

**Table 4:** Percentage of correct answers by cSLE patients in each subtest of the Ped-ANAM battery and comparison with healthy controls.

Subtest	Min–Max	1Q–3Q	Median	*p*-Value
Simple reaction time	100	100	100	0.2437
Matching to sample	30–100	60–95	0.85	0.4526
Spatial processing	50–100	85–95	0.90	0.7607
Memory search	22–100	73–94	0.86	0.2733
Mathematical processing	30–100	80–100	0.90	0.8809
Matching grids	30–100	90–100	0.95	0.2681
Code substitution	45–100	93–98	0.97	0.0065 *
Logical relations	55–100	95–100	100	0.0719

cSLE, childhood-onset SLE; Ped-ANAM, Pediatric Automated Neuropsychological Assessment Metrics, Min–Max, minimum–maximum percentage of correct answers; 1Q, first quadrant, 3Q, third quadrant. Mann–Whitney U test; * Significant *p* value ≤ 0.05.

**Table 5:** Percentage of correct answers by aSLE patients in each subtest of the Ped-ANAM battery and comparison with healthy controls.

Subtest	Min–Max	1Q–3Q	Median	*p*-Value
Simple reaction time	0–100	100–100	100	0.1440
Matching to sample	30–100	85–95	85	<0.001 *
Spatial processing	40–100	90–95	90	0.0036
Memory search	15–100	65–92	86	<0.001 *
Mathematical processing	30–100	95–100	90	0.040 *
Matching grids	40–100	90–100	95	0.0080 *
Code substitution	45–100	98–100	98	0.0157 *
Logical relations	50–100	95–100	100	0.0129 *

aSLE, adult-onset SLE; Ped-ANAM, Pediatric Automated Neuropsychological Assessment Metrics; Min–Max, the minimum–maximum percentage of correct answers; 1Q, First quadrant, 3Q, Third quadrant. Mann–Whitney U test; * Significant *p* value ≤ 0.05.

**Table 6:** Descriptive analysis of the PVI of all participants in each Ped-ANAM subtest (N = 378).

	SLE Patients (N = 201)	Healthy Controls (N = 177)	*p*-Value	cSLE Patients (N = 41)	Healthy Controls (N = 63)	*p*-Value	aSLE Patients (N = 160)	Healthy Controls (N = 114)	*p*-Value
Median	7	2	<0.001 *	4	2		7	2	
Min–Max	0–33	0–22		0–23	0–18	0.0271 *	0–33	0–22	<0.001 *
PVI >14 N (%)	38 (18.9%)	8 (4.5%)		8 (19.5%)	3 (4.7%)		32 (20%)	4 (3.5%)	

cSLE, childhood-onset SLE; aSLE adult-onset SLE; Min–Max, minimum–maximum. Mann–Whitney U test; * Significant *p* value ≤ 0.05.

**Table 7:** Descriptive clinical outcomes in aSLE and cSLE (N = 201).

		cSLE Patients (N = 41)	aSLE Patients (N = 160)	*p*-Value
SLEDAI	Median	0	5	0.057
	Min–Max	0–10	0–16	
SDI	Min–Max	0–4	0–4	<0.0001 *
	>1 N (%)	1 (2.43%)	59 (31.05%)	
NPSLE	Presence N (%)	19 (10.0%)	49 (25.8%)	0.0576
	Absence N (%)	22 (12%)	111 (54.2%)	

cSLE, childhood-onset SLE; aSLE, adult-onset SLE; SLEDAI, Systemic Lupus Erythematosus Disease Activity Index; SDI, Rheumatology Damage Index; NPSLE, Neuropsychiatric Systemic Lupus Erythematosus; Min–Max, minimum–maximum; SDI, <0.0001 * aSLE had a greater difference in distribution.

## Data Availability

The raw data supporting the conclusions of this article will be made available by the authors, without undue reservation.
